# *FGFR3* mutation frequency in 324 cases from the International Skeletal Dysplasia Registry

**DOI:** 10.1002/mgg3.96

**Published:** 2014-08-05

**Authors:** Yuan Xue, Angela Sun, P Betty Mekikian, Jorge Martin, David L Rimoin, Ralph S Lachman, William R Wilcox

**Affiliations:** 1 Department of Human Genetics, Emory UniversityAtlanta, Georgia, 30322; 2 Medical Genetics Institute, Cedars-Sinai Medical CenterLos Angeles, California; 3 Department of Pediatrics, UCLA School of MedicineLos Angeles, California

**Keywords:** Achondroplasia, FGFR3, hypochondroplasia, mutation frequency, thanatophoric dysplasia

## Abstract

Fibroblast growth factor receptor 3 (*FGFR3*) is the only gene known to cause achondroplasia (ACH), hypochondroplasia (HCH), and thanatophoric dysplasia types I and II (TD I and TD II). A second, as yet unidentified, gene also causes HCH. In this study, we used sequencing analysis to determine the frequency of *FGFR3* mutations for each phenotype in 324 cases from the International Skeletal Dysplasia Registry (ISDR). Our data suggest that there is a considerable overlap of genotype and phenotype between ACH and HCH. Thus, it is important to test for mutations found in either disorder when ACH or HCH is suspected. Only two of 29 cases with HCH did not have an identified mutation in *FGFR3*, much less than previously reported. We recommend testing other mutations in *FGFR3*, instead of just the common HCH mutation, p.Asn540Lys. The mutation frequency for TD I and TD II in the largest series of cases to date are also reported. This study provides valuable information on *FGFR3* mutation frequency of four skeletal dysplasias for clinical diagnostic laboratories and clinicians.

## Introduction

Achondroplasia (ACH; MIM:100800) occurs with an estimated prevalence between 1/16,000 and 1/26,000 live births, representing the most common genetic form of human dwarfism (Orioli et al. 1995). Affected patients have short limbs with macrocephaly and characteristic facial features such as frontal bossing and midface hypoplasia. A milder form of ACH is hypochondroplasia (HCH, MIM: 146000). Thanatophoric dysplasia (TD) (MIM: 187600), with an incidence between 1/33,000 and 1/47,000 live births (Waller et al. 2008), is the most common form of neonatal lethal dwarfism. Individuals usually die after birth from respiratory distress secondary to pulmonary hypoplasia. Two phenotypes have been distinguished: the more common form with curved femora (TD I) or the less frequent form with straight femora and a cloverleaf skull (TD II) (Wilcox et al. 1998). Fibroblast growth factor receptor 3 (*FGFR3,* OMIM# 134934) is the only known gene associated with all four of these skeletal dysplasias as well as severe ACH with developmental delay and acanthosis nigricans (SADDAN), Crouzon syndrome with acanthosis nigricans (MIM: 612247), and Muenke craniosynostosis syndrome (MIM: 602849) (Bellus et al. 1999; Mulliken et al. 1999; Zankl et al. 2008).

FGFR3 is a transmembrane tyrosine kinase receptor that binds fibroblast growth factors. The *FGFR3* gene, located on chromosome 4p16.3 (Thompson et al. 1991), consists of 18 coding exons (Keegan et al. 1991, 1993). It was first linked to ACH by Shiang et al. (1994) and Rousseau et al. (1994). Mutations in *FGFR3* causing skeletal dysplasia are all inherited in an autosomal dominant pattern, but frequently occur de novo on the paternal allele (Wilkin et al. 1998). The mutations are gain of function causing increased activation of FGFR3 with the severity of the phenotype proportionate to the overactivation of the receptor. FGFR3 overactivation slows endochondral ossification in part through ERK/MAPK (mitogen-activated protein kinases, originally called ERK, extracellular signal-regulated kinases; Foldynova-Trantirkova et al. 2012). Loss of FGFR3 function causes autosomal recessive camptodactyly, tall stature, and hearing loss (CATSHL syndrome; MIM: 610474).

Two mutations in the *FGFR3* gene at nucleotide c.1138 (most commonly a G-to-A transition with a less frequent G-to-C transversion) causing a p.Gly380Arg substitution are said to account for the majority of ACH (Shiang et al. 1994; Bellus et al. 1995a,b). Mutations causing HCH are more widespread in *FGFR3* with a hotspot in the tyrosine kinase domain at codon 540 in exon 13 (Bellus et al. 1995a,b). Other rare HCH mutations have been reported (Bellus et al. 2000; Heuertz et al. 2006). However, a significant number of HCH cases have no mutations in *FGFR3*, indicating that other genes may be involved in this phenotype (Flynn and Pauli 2003). Several amino acid substitutions in the extracellular and intracellular domains of the *FGFR3* protein have been found in TD I, including p.Arg248Cys, p.Tyr373Cys, and p.Lys650Met (Tavormina et al. 1995; Rousseau et al. 1996a,b,c). In addition, several mutations in the stop codon mutations have also been described (Rousseau et al. 1996a,b,c). Platyspondylic lethal skeletal dysplasia, San Diego type (PLSD-SD) (MIM# 187600), shares many phenotypic features with TD I and is due to the same *FGFR3* mutations as TD I (Brodie et al. 1999). For TD II, only the p.Lys650Glu mutation in *FGFR3* has been found (Wilcox et al. 1998). In this study, conducted since 1994, we investigated the frequency of mutations in clinically and radiographically diagnosed cases of ACH, HCH, TD I and II from the International Skeletal Dysplasia Registry (ISDR).

## Methods

For the last 40 years, samples have been received through the ISDR (http://cedars-sinai.edu/Patients/Programs-and-Services/Skeletal-Dysplasia/). These cases were referred to us by clinicians around the globe and seen in our own clinics. The diagnoses were based on established clinical, radiographic, and for many TD cases, chondro-osseous features. For the purpose of this study, we have combined TD I and PLSD-SD, since they are due to similar mutations in *FGFR3* (Brodie et al. 1999). These cases include 93 cases of TDI + PLSD-SD (Tavormina et al. 1995; Brodie et al. 1998, 1999; Kitoh et al. 1998; Wilcox et al. 1998) and 17 cases of TDII (Tavormina et al. 1995; Wilcox et al. 1998) that were previously reported. Almost all cases were referred to the ISDR without prior molecular testing. We excluded cases that had undergone commercial testing and identified a mutation. The study is approved by Cedars-Sinai Medical Center human subjects Institutional Review Board.

Genomic DNA was isolated from samples (whole blood, frozen tissue, or cultured cells) using a commercial kit (Qiagen). All the DNA samples were first tested for the mutations commonly seen for each disorder in *FGFR3* (ACH and HCH: p.Gly380Arg and p.Asn540Lys; TD I: p.Arg248Cys, p.Tyr373Cys, p.Ser249Cys, and p.X807 mutations; TD II: p.Lys650Glu) using Sanger sequencing. Sequencing of all the coding exons was performed if no common mutations were found. For primer's sequences see Table S1 (Reference sequence for the *FGFR3* gene: NM_000142.4). Polymerase chain reaction (PCR) conditions were as follows: denaturing at 94°C; annealing at 64°C for 5 cycles, 62°C for 5 cycles, and 60°C for 25 cycles; elongating at 72°C; amplification for 30 cycles. PCR products from the more recent cases were directly sequenced using the Big Dye Sequencing kit (Applied Biosystems, Foster City, CA) on an ABIPRISM 3130 Genetic Analyzer and analyzed with the Sequencing 5.2 software package (Applied Biosystems). Older cases were analyzed by prior ABI machines or gel electrophoresis. Radiographs and clinical information from patients carrying *FGFR3* mutations uncommon to the phenotype were reexamined after mutation detection to verify the phenotypic assignment was correct.

## Results

The frequency of *FGFR3* mutations in four skeletal dysplasias is shown in Table1. About 90% of ACH cases had a *FGFR3* p.Gly380Arg mutation with the majority due to a c.G1138A substitution. The remainder of the cases had a p.Asn540Lys mutation due to one of two c.1620 substitutions. For HCH, 75.9% of cases had a p.Asn540Lys mutation. p.Gly380Arg and p.Lys650Gln mutations each account for 6.9% of the HCH cases. We found one case with a rare p.Tyr278Cys mutation. Two HCH cases did not have an identified *FGFR3* mutation.

**Table 1:** Mutation frequency for different skeletal dysplasia phenotypes

Phenotype	Total No. of cases	Protein amino acid change	cDNA Nucleotide change	No. of cases	Percentage (%)
ACH	79	p.Gly380Arg	c.1138G>A	58	89.9
			c.1138G>C	13	
		p.Asn540Lys	c.1620C>A	5	10.1
			c.1620C>G	3	
HCH	29	p.Tyr278Cys	c.829A>G	1	3.4
		p.Gly380Arg	c.1138G>A	2	6.9
		p.Asn540Lys	c.1620C>A	13	75.9
			c.1620C>G	9	
		p.Lys650Gln	c.1949A>C	2	6.9
		No mutation	N/A	2	6.9
TD I* (includes PLSD-SD)	173	p.Arg248Cys	c.742C>T	115	66.5
		p.Ser249Cys	c.746C>G	11	6.4
		p.Gly370Cys	c.1108G>T	4	2.3
		p.Tyr373Cys	c.1118A>G	41	23.7
		p.Lys650Met	c.1949A>T	2	1.2
		p.X807Arg	c.2419T>A	2	6.9
		p.X807Arg	c.2419T>C	2	
		p.X807Gly	c.2419T>G	3	
		p.X807Ser	c.2420G>C	1	
		p.X807Leu	c.2420G>T	1	
		p.X807Trp	c.2421A>G	3	
TD II*	31	p.Lys650Glu	c.1948A>G	31	100.0

Reference sequence for *FGFR3*: NM_000142.4. Phenotype symbols: ACH, achondroplasia; HCH, hypochondroplasia; TDI, thanatophoric dysplasia I; TDII, thanatophoric dysplasia II.

* Includes 93 cases of TDI + PLSD-SD (Tavormina et al. 1995; Brodie et al. 1998, 1999; Kitoh et al. 1998; Wilcox et al. 1998) and 17 cases of TDII (Tavormina et al. 1995; Wilcox et al. 1998).

The p.Arg248Cys mutation caused the majority (66.5%) of the TD I causes, followed by p.Tyr373Cys. The third most common mutation for TD I was at the stop codon p.X807, which was closely followed by the p.Ser249Cys mutation. p.Gly370Cys and p.Lys650Met accounted for a minority of cases, 2.3% and 1.2%, respectively. The p.Lys650Glu mutation was exclusively found in all cases of TD II.

**Figure 1:**
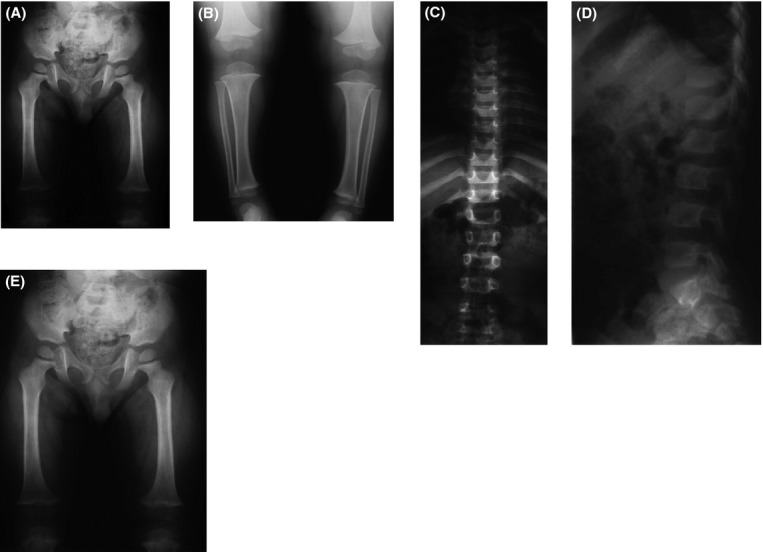
Radiographs of a case of achondroplasia with a p.Asn540Lys mutation (R#00-347). (A) Pelvis and femur, age 3 years. Narrow sacrosciatic notches and femoral shortening. (B) AP lower legs, age 3 years. There is distal overgrowth of the fibulae. (C) AP spine, age 3 years, shows platyspondyly and interpediculate narrowing in the lumbar spine. (D) Lateral spine, age 3 years. Note a narrow spinal canal and a wedge vertebrae at L2. (E) PA, left hand, age 3 years. There is significant brachydactyly and brachymetacarpia.

Figure1 shows the radiographs for one of the patients with clinical ACH due to a p.Asn540Lys mutation (R#00-347). He was first seen at our center at 40 months of age. He was brought in by his adoptive parents who knew little of his prior history. Born at 32 weeks, he was diagnosed with ACH in the first year of life. His height was 85 cm (+2 SD on the ACH growth chart) and head circumference 53 cm (95%). He had macrocephaly, midface hypoplasia, rhizomelic shortening of the limbs, and brachydactyly typical for ACH. At his last visit, he was 11.5 years old and his height was 132.8 cm (+2.5 SD on the ACH chart) and his head circumference was 55.9 cm (95%). He had developed genu valgum in addition to the physical features noted previously.

Radiographic findings, such as narrow spine canal, a wedge vertebrae at L2, (Fig.1D) and significant brachydactyly and brachymetacarpia, support a diagnosis of ACH.

**Figure 2:**
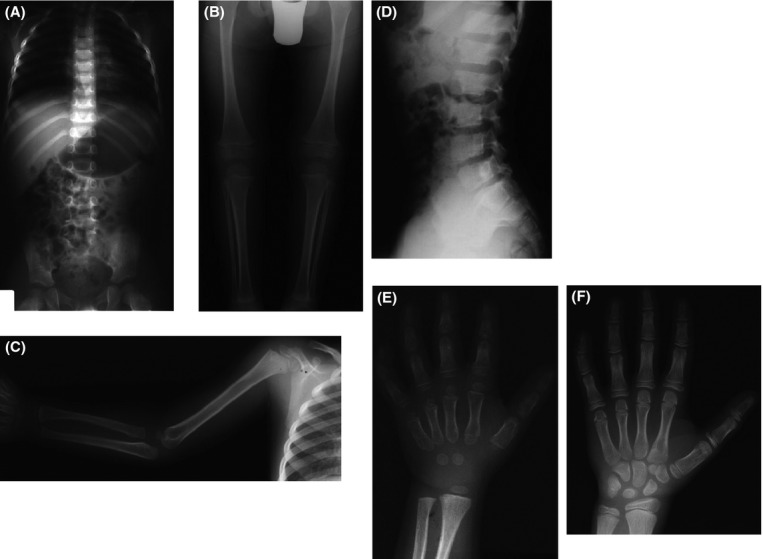
Radiographs of a case of hypochondroplasia with a p.Gly380Arg mutation (R# 92-231A). (A) AP chest and pelvis, age 5 years. The sacrosciatic notch is narrowed, but there is no interpediculate narrowing. (B) AP legs, age 5 years. There is distal fibular overgrowth. (C) AP arm, age 5 years. There is only mild rhizomelic shortening. (D) Lateral lumbar spine, age 12.5 years. There is mild platyspondyly and a narrowed spinal canal. (E) PA left hand, age 5 years. Note the brachydactyly and brachymetacarpia. (F) PA, left hand, age 12.5 years. The brachymetacarpia is less pronounced than at age 5.

In Figure2, milder radiographic findings such as mild interpediculate narrowing suggest a diagnosis of HCH although this patient (R#92-231A) had a p.Gly380Arg mutation. He was born at term and his growth was in the normal range until age 2. The paternal height was 188 cm and the maternal height was 163 cm. At age 6 years 9 months, he began being followed by an endocrinologist. His height was 105.8 cm (+2.5 SD on the ACH chart). He had rhizomelic shortening of the limbs and mild midface hypoplasia without macrocephaly. At age 10 years 10 months, his height was 125.7 cm (+2.5 SD on the ACH chart). At 12 years 7 months, he was 131.8 cm tall (+2 SD on the ACH chart). He was placed on growth hormone therapy at 12.5 years.

## Discussion

### Overlapping genotype and phenotype of ACH and HCH

Both ACH and HCH share clinical and radiological features including macrocephaly, brachydactyly, metaphyseal flaring, narrowing of the interpediculate distance in the lumbar spine, square iliae, and short femoral necks. The abnormalities seen in HCH are less severe overall than those seen in ACH and HCH patients have less dysmorphic facial features (Matsui et al. 1998). Most cases can be readily distinguished clinically and radiographically. However, there is variability in severity within each group, which sometimes makes it difficult to differentiate severe HCH from mild ACH cases. Based on our large series of cases, the frequency of *FGFR3* mutations in ACH and HCH is considerably different than that previously reported. It has been claimed that two common mutations in *FGFR3,* both resulting in p.Gly380Arg amino acid substitutions, cause over 99% of ACH cases (Shiang et al. 1994; Bellus et al. 1995a,b; Rousseau et al. 1996a,b,c). In this study, 10% of ACH is caused by p.Asn540Lys that is more typically associated with HCH. Interestingly, the p.Gly380Arg mutation was found in about 7% of the clinically diagnosed HCH cases. These data indicate that there is an overlap in the phenotypic spectrum of the p.Gly380Arg and p.Asn540Lys mutations. In our series, we did not identify some of the mutations found in other cohorts, such as p.Asn540Thr (Deutz-Terlouw et al. 1998), p.Asn540Ser (Mortier et al. 2000), p.Ile538Val (Grigelioniene et al. 1998), and p.Asn328Ile (Winterpacht et al. 2000).

In clinical practice, molecular tests are normally used for confirmatory purposes. Most laboratories offer targeted mutation analysis separately for ACH and HCH. Testing for ACH consists of the two point mutations for p.Gly380Arg, whereas testing for HCH is usually limited to p.Asn540Glu. Given the overlapping mutations between these two skeletal dysplasias, we recommend that laboratories should include both p.Gly380Arg and p.Asn540Glu mutations on the test menu when either ACH or HCH is suspected. This study provides data from the first and largest series of cases to support this testing strategy. Until laboratories adopt this practice, clinicians should consider ordering both tests. With this strategy, almost all cases of ACH will have a detected mutation while the detection rate for HCH will be ∼80%.

### Incomplete screening may explain the 70% detection rate for FGFR3 mutations in HCH

It has been reported that about 30% of HCH cases do not have a mutation in *FGFR3* (Prinos et al. 1995; Bellus et al. 1996; Rousseau et al. 1996a,b,c; Fofanova et al. 1998; Prinster et al. 1998; Ramaswami et al. 1998). We found that p.Asn540Lys, p.Gly380Arg, and p.Lys650Gln mutations in *FGFR3* together account for about 90% of the cases. The p.Asn540Lys mutation alone accounts for about 76%, which is in agreement with other studies for HCH (Prinos et al. 1995; Bellus et al. 1996; Rousseau et al. 1996a,b,c; Fofanova et al. 1998; Prinster et al. 1998; Ramaswami et al. 1998). A less common mutation in *FGFR3*, p.Lys650Gln, was found in 7.4% of HCH cases. This mutation has been reported before with a slightly milder skeletal phenotype than found with the p.Asn540Lys mutation (Bellus et al. 2000). The p.Tyr278Cys mutation, found in one HCH patient, has been reported previously in two patients (Heuertz et al. 2006). These patients had an ACH phenotype at birth, at the age of 6 months, and during the first 2 years of life. By the age of 3.5 years, the phenotype had changed to typical HCH with normal craniofacial features. Our patient is an adult who had HCH clinically and radiographically. It is possible that this patient had an ACH phenotype at younger age and evolved to HCH as he grew older.

The much higher detection rate observed in this study suggests that failing to look for other mutations in *FGFR3* such as p.Gly380Arg and p.Lys650Gln maybe the reason for the ∼70% detection rate quoted for HCH. Since all mutations in *FGFR3* causing dwarfism are activating mutations, it is not likely that sequencing the coding region will miss a pathogenic mutation. Thus, from our data, genetic locus heterogeneity is to be found in less than 10% of HCH cases. With advanced sequencing technology such as exome sequencing, it is highly possible that the second HCH locus will be identified in the future.

### What to expect when testing mutations for TD I and TD II

Although several distinct missense mutations have been described for TD I cases, the most frequent mutations are p.Arg248Cys and p.Tyr373Cys, these two mutations together contributing to about 90% of the cases. TD II patients exclusively have p.Lys650Glu mutation, which agrees with previous studies (Wilcox et al. 1998; Bellus et al. 2000). This information is based on the largest series of TD cases and can help clinical laboratories design a mutation panel for TD, especially for prenatal diagnosis.

### Limitations of the study

Some cases (<10%) were referred to the ISDR with known mutations from clinical laboratories. These cases were not analyzed in this study and are excluded from this analysis. We included only cases where we did the molecular analysis. Because commercial testing typically only tests for the common mutations for each phenotype and we sequenced the entire coding region, if necessary, the percentage of rare mutations in a completely unselected population could be slightly lower than we found.

## Conclusion

Based on a large number of cases, we report the mutation frequency in *FGFR3* for four major skeletal dysplasias. This information can be used to significantly improve analytical sensitivity in a clinical molecular laboratory. When considering a testing strategy, either a mutation panel or reflex testing could be used for ACH and HCH. Panels can be designed to test all these mutations simultaneously. Otherwise, reflex testing can be applied after the common mutation is not detected for a specific phenotype. For example, a p.Asn540Lys mutation should be considered when a p.Gly380Arg mutation is not found in a suspected case of ACH patient and vice versa for HCH. For TD II, p.Lys650Glu is the only mutation that needs to be tested for. In TD I, p.Arg248Cys and p.Tyr373Cys would be tested first followed by less common mutations.

## Supporting Information

Table S1 Sequences of primer pairs used to amplify *FGFR3* coding exons.
