# Supplementary material for: FGFR3 mutation frequency in 324 cases from the International Skeletal Dysplasia Registry
Source: Mol Genet Genomic Med. 2014 Aug 5;2(6):497–503. doi: 10.1002/mgg3.96 (PMC4303219; doi:10.1002/mgg3.96)
Supplement: Table S1 — Sequences of primer pairs used to amplify FGFR3 coding exons. [file mgg30002-0497-sd1.docx]

Supplementary Table. Sequences of primer pairs used to amplify *FGFR3* coding exons.

| Exon | Primer Sequence (5' --> 3') |
| --- | --- |
| 2 F | AGGGGTCGGGACGCAGGAG |
| 2 R | CCCAACGCCTCTGCCCGCAC |
| 3 F | GTCTGTAAACGGTGCCGG |
| 3R | ACCAGAGAGACCCCCAGC |
| 4 F | ATCTGGGAGGGGCACCTGGG |
| 4 R | GTCCCTCAGCTGCCTGTGAAG |
| 5 F | GTTCAGAGGGGCCTCTGCTC |
| 5 R | AGTGAGCGGAGGCAGCAACC |
| 6 F | CAGACGCGGTGGTTGCTGCC |
| 6 R | GCACGTCCAGCGTGTACGTCTG |
| 7 F | CGGCAGTGGCGGTGGTGGTG |
| 7 R | CCAGCCCAGGAGCCCCAGCG |
| 8 F | TCTCCCACATCCTGCCTC |
| 8 R | GGGCCTTGGAGCTGGAGCTC |
| 9 F | AGGGCGGTGCTGGCGCTCGC |
| 9 R | AGACAGTGCGGAGCAGCAGC |
| 10 F | CCTCAACGCCCATGTCTTT |
| 10 R | AGGCAGCTCAGAACCTGGTA |
| 11 F | CTGTACCTCCACGCCCTGTCGC |
| 11 R | CTGTTTCACCCCCACCACC |
| 12 F | GAGTGGGCGAGTTTGCACACTC |
| 12 R | GCCCCCAGCCCTGCTCTGCAC |
| 13 F | GTGCAGAGCAGGGCTGGGGGC |
| 13 R | GCTCCTCAGACGGGCTGCCAG |
| 14 F | CTGGCAGCCCGTCTGAGGAGC |
| 14 R | CTGCTCCCAGCATCTCAGGGCA |
| 15 F | GGTGGAGAGGCTTCAGCCCT |
| 15 R | GCCAGGCGTCCTACTGGCATGA |
| 16 F | TCATGCCAGTAGGACGCCTGGC |
| 16 R | GGTCCTGGCTCTGCCCAGTTC |
| 17 F | CAGCGCAGCCCTGGCCTATT |
| 17 R | CCTGAAGGGCTGCCAGTCCCT |
| 18 F | GAAGCGGCGGGGCTCACTCCT |
| 18 R | ATAGGCGGGTGGCACCAGGC |
| 19 F | GCGAAGAGGGGCTCGGTGGCAC |
| 19 R | CACCAGCAGCAGGGTGGGCTGCTAG |
